# Culture and Real-Time PCR Based Maternal Screening and Antibiotic Susceptibility for Group B Streptococcus: An Iranian Experience

**DOI:** 10.5539/gjhs.v7n6p233

**Published:** 2015-04-16

**Authors:** Gholamreza Goudarzi, Masoumeh Ghafarzadeh, Pegah Shakib, Khatereh Anbari

**Affiliations:** 1 School of Medicine, Lorestan University of Medical Sciences, Khorramabad, Iran

**Keywords:** antibiotic susceptibility, group B streptococcus, pregnant women, real time-PCR

## Abstract

**Introduction:**

Vertical Transmission of group B streptococcus (GBS) from a vagina colonized mother to her infant upon rupture of membranes (ROM) or after the onset of labor can cause life-threatening infections in newborn. Although intrapartum antibiotic prophylaxis (IAP) can significantly decrease neonatal GBS diseases, this issue has potentiated the emergence of antibiotic resistance strains. Our study examined the colonization rate of GBS using real-time PCR and culture methods, and trends in antibiotic resistance of GBS isolates obtained from pregnant women in Khorramabad, Iran.

**Methods:**

In this cross-sectional study, two vaginal-rectal swabs were collected and analyzed separately from 100 pregnant women at 35-37 weeks of gestation by convenience sampling method. The specimens were subjected to GBS detection using real-time PCR assay and standard culture. Susceptibility pattern of the GBS isolates was examined using the disk diffusion method.

**Results:**

GBS carriage rate was 17% and 19% using culture and real-time PCR, respectively. In six samples, the culture was positive and the real-time PCR was negative. Sensitivity and specificity for real-time PCR were 72.7% and 96.1%, respectively using culture as the gold standard. Amongst twenty-two isolates examined, 100% resistance to erythromycin and clindamycin was observed. One isolate (4%) exhibited resistance to penicillin.

**Conclusion:**

Considering the relatively high GBS carriage rate in Khorramabad, routine antepartum screening for GBS is recommended. Penicillin can remain the antibiotic of choice for IAP; however, in penicillin-allergic mothers, vancomycin can be an alternative antibiotic.

## 1. Introduction

Vertical transmission of group B streptococcus (GBS) from a vagina colonized mother to her infant upon rupture of membranes (ROM) or after the onset of labor can cause life-threatening infections in newborn (Jolivet, 2002). According to the Centers for Disease Control and Prevention (CDC) guidelines, universal antepartum screening of pregnant women at 35-37 weeks of gestation and subsequent intrapartum antibiotic prophylaxis (IAP) of rectal and/or vaginal colonized women, has increased the concern regarding antibiotic resistance development; so that, in some study, increased resistance to first choice antibiotics, such as penicillins and macrolides have been demonstrated (Heelan, Hasenbein, & McAdam, 2004; Kimura et al., 2008; Simoes, Aroutcheva, Heimler & Faro, 2004; Verani, McGee & Schrag, 2010). Although cultivation of clinical specimens and emergence of bacterial colonies is still the gold standard to detect various bacteria and it provides the conditions for the assessment of antimicrobial susceptibility of isolates; nevertheless, culture and traditional phenotypic identification are difficult and time-consuming. Therefore, through the past decade, several studies have focused on rapid and reliable methods such as immunoassay, hybridization techniques, and nucleic acid amplification (NAA) technology, especially polymerase chain reaction (PCR) and real-time PCR (de-Paris, Machado, Gheno, Ascoli & Oliveira, 2011; Gavino & Wang, 2007; Honest et al., 2006; Peltroche-Llacsahuanga, Fiandaca, Oy, Lutticken & Haase, 2010). In Iran, including Khorramabad (west of Iran), screening of pregnant women for GBS has not been performed and there is rare reported regional information about GBS frequency rate and its susceptibility pattern. Therefore, the aim of this study was to determine the incidence of maternal GBS colonization by using culture and real-time PCR methods and also to assess the antibiotic susceptibility profile of isolated GBS collected from pregnant women attending the obstetrics and gynecology clinics of Khorramabad, Iran.

## 2. Methods

### 2.1 Samples Collection and GBS Screening Culture

In this cross-sectional study, one-hundred pregnant women at 35-37 weeks of gestation, attending selected gynecology and obstetrics clinics (referral centers) of Khorramabad, were enrolled from April to July 2012. The sampling method was convenience sampling. The minimum required sample size was calculated based on the formula for calculating a proportion. Two rectal and two vaginal swabs were collected from patients by a trained gynecologist. One of the rectal and vaginal swabs were separately transferred into tubes containing 2 ml of Todd-Hewitt broth (Merck, Germany) supplemented with nalidixic acid (15μg/ml, Sigma) and gentamicin (8μg/ml, Sigma). Selective broth mediums were then incubated for approximately 18 h at 37°C before sub-culturing to 5% sheep blood agar. Inoculated plates were further incubated at 37°C in 5% CO_2_ for 24-48 h. Presumptive streptococcal colonies were targeted to further identification using standard microbiology methods include: gram staining, catalase, CAMP (Christie-Atkins-Munch-Petersen) and hippurate hydrolysis tests (Baron, 2003; Larsen & Sever, 2008; Lehman, Mahon & Suvarna, 2011).

### 2.2 Susceptibility Testing

All identified GBS isolates were tested for susceptibility to penicillin, erythromycin, clindamycin and vancomycin using bacterial suspension equivalent to 0.5 McFarland standard. The Clinical and Laboratory Standards Institute (CLSI) recommended procedures for disk susceptibility testing were used on blood agar bacterial culture at 35°C in a 5% CO_2_ atmosphere (Clinical and Laboratory Standards Institute, 2011).

### 2.2 DNA extraction and Real-Time PCR Assay

In order to extract DNA, the other rectal and vaginal swabs were separately placed into tubes containing 1 ml sterile phosphate buffered saline (PBS). The tubes were vigorously vortexed for one minute and then swabs were discarded. After centrifugation of tubes and removing the supernatant, the cell precipitate was well resuspended in 200 µl of lysis solution [containing 10µl lysozyme (Merck; 20 mg/ml) and 190 µl PBS]. The tubes were further incubated at 37°C for 30 minutes (Bergh, Stoelhaug, Loeseth & Bevanger, 2004). The DNA samples were extracted using *AccuPrep*^®^ Genomic DNA Extraction Kit (Bioneer, Korea) according to manufacturer’s instructions. The samples were stored at -20°C until the experiments. A commercial real-time PCR kit (Quantification of *Streptococcus agalactiae*, PrimerDesign™ Ltd.UK) was used to detect GBS-specific DNA from the swab samples. GBS was identified using specific DNA primer/probe mix (FAM labeled, BHQ quenched) designed to detect *cfb* gene (CAMP factor). To ensure extraction of the valid biological templates and the lack of PCR inhibitors, this kit contains a separate primer/probe mix to detect the Actin Beta (ACTB) gene as internal control, as well as, the GBS genome as a positive control. This assay was performed in a final volume of 20 µl containing 10 µl mastermix (Primer Design Precision™ 2X qPCR Mastermix), 1 µl primer/probe mix, 3 µl (25 ng) template and 6 µl RNAse/DNAse free water. Cycling parameters were optimized and run according to the manufacturer’s protocol with a little modification on a Rotor Gene thermocycler (Corbett RG-6000). Samples that showed negative internal control results were re-extracted or diluted before repeat.

### 2.2 Statistical Analysis

Sensitivity, specificity, positive predictive value (PPV) and negative predictive value (NPV) for the real-time PCR technique with 95% confidence interval were calculated using enriched culture as the gold standard. Chi-square test was used to determine any significant difference between results of real-time PCR technique and enriched culture.

### 2.5 Ethical Considerations

This study was approved by the ethics committees of Lorestan University of Medical Sciences, Khorramabad, Iran. Written informed consents were obtained from all pregnant women included in the study.

## 3. Results

**Figure 1:**
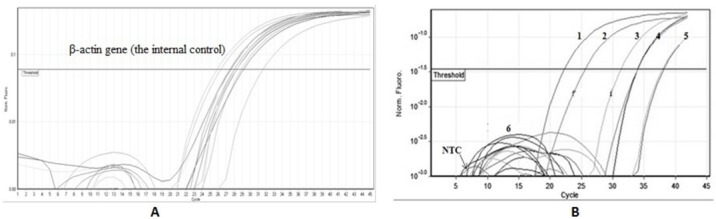
Real time-PCR based detection GBS in rectal/vaginal specimens collected from 100 pregnant women

Based on culture results, 17 pregnant women (17%) were identified as antenatal carrier for GBS in vagina (n = 9), rectum (n = 3) or in both (n = 5). In other words, 22 GBS isolates were obtained from the specimens cultured from 100 tested pregnant women. Whereas, according to the real-time PCR results, 19% of participants were (9 only vaginally, 6 only rectally, and 4 both vaginally and rectally) were colonized for GBS. The amplification curves for β-actin gene (the internal control) effectively demonstrated DNA extraction from all swab samples and the lack of PCR inhibitors (Figure 1A). Representative curves for the *cfb* gene of templates containing GBS DNA in various samples are also presented in Figure 1B.

(A) Amplification curves to detect **β**-actin gene (the internal control) for some swab samples; (B) representative curves to detect templates containing GBS DNA in various rectal/vaginal specimens. 1, positive control (pure DNA of GBS); 2-5, positive rectal/vaginal swab samples; 6, negative swab specimen; NTC, no template control (distillated water). GBS was identified using specific primer/probe mix (FAM labeled, BHQ quenched) designed to detect *cfb* gene (CAMP factor).

The results of culture and real-time PCR for GBS in rectal and vaginal samples are compared in Table 1. As seen, five of the tested samples had a positive vaginal culture; while their PCR results were negative; amongst them, one subject had simultaneously positive results vaginally and rectally by culture; while negative PCR results for both samples were yielded.

**Table 1:** Comparison between pre-enriched culture and real-time PCR for detection of GBS in vaginal and/or rectal swabs in 100 pregnant women at 35-37 weeks of gestation

	Real time PCR +		Real time PCR -		P-value
	
	Vaginal	Rectal	Vaginal	Rectal	
Culture +	9	7	5	1	<0.001
Culture -	4	3	82	89	

In our study, the real time PCR technique to detect GBS among the swab specimens from pregnant women has a sensitivity of 72.7% [95% confidence interval (CI) 0.64–0.814] and a specificity of 96.1% (95% CI 0.924–0.998), when using culture as the gold standard (Table 2).

**Table 2:** Characteristics of sensitivity and specificity of real-time PCR compared with culture method (as the gold standard) for detection of GBS in 200 recto-vaginal swab specimens

Real-time PCR Culture	Positive	Negative
Positive	16	6
Negative	7	171
Sensitivity	72.7%	
95% CI	64 - 81.4%	
Specificity	96.1%	
95% CI	92.4 - 99.8%	
PPV^*^	69.6%	
95% CI	63.2 -76%	
NPV ^*^	96.6%	
95% CI	93.1- 99.9%	
False positive	3.9%	
False negative	27.3%	

* PPV, positive predictive value; NPV, negative predictive value.

The disk diffusion method for antimicrobial susceptibility testing showed all of the 22 isolates tested obtained from cultured vaginal/rectal specimens, to be sensitive to vancomycin. Whereas, 21 isolates (95.45%) were sensitive to penicillin, one isolate (4.5%) was resistant. Notably, all tested isolates were resistant to both antibiotics erythromycin and clindamycin.

## 4. Discussion

This study reports the prevalence of GBS carriage in a limited population of pregnant women using two different GBS screening methods, as well as, the pattern of antibiotic susceptibility for yielded isolates. Based on culture results, GBS was present in the vagina or/and rectum of 17% of the tested pregnant women. This is consistent with GBS infection rates reported from various countries of the world (10.0%-34.7%) (Arisoy, Altinisik, Tünger, Kurutepe, & Ispahi, 2003; Bergh et al., 2004; Bergseng, Bevanger, Rygg & Bergh, 2007; de-Paris et al., 2011; Faro et al., Volume 2010; Goodrich & Miller, 2007). Also, in two studies in Tehran (Iran), Bakhtiari et al.(Bakhtiari, Soltan Dallal, Fallah Mehrabadi, Heidarzadeh & Pourmand, 2012) and Javanmanesh et al. (Javanmanesh & Eshraghi, 2013), the GBS carriage rate found 9.3% and 22.76%, respectively. Out of 200 swab samples collected in the current study, 16 (rectal or vaginal) tested samples were positive for GBS by both culture and real-time PCR. Seven samples had positive real-time PCR results, while negative culture results were identified (Table 1). Although these positive results are statistically considered as false-positive when they are compared to the culture results (as the gold standard), GBS may really exist. In other words, the use of antibiotics, antimicrobial sanitizing agents, over growth of intestinal or vaginal bacteria and even light bacterial colonization might have reduced the sensitivity of culture to detect positive subjects and so yielded negative results. In the present study, the sensitivity and PPV of real-time PCR (versus to culture method) were 72.7% and 69.6%, respectively (Table 2). These values represented lower sensitivity of this test in comparison with the rates reported by previous studies (92.5%-97.0%) (Bergseng et al., 2007; Gavino & Wang, 2007; Goodrich & Miller, 2007). Nevertheless, real-time PCR demonstrated to have high specificity (96.1%) and NPV [(96.6%), (Table 2)], which is consistent with the values found by corresponding studies (64.5%-99.0%) (Bergseng et al., 2007; Gavino & Wang, 2007; Goodrich & Miller, 2007). The high specificity of real-time PCR is an important finding of our study. It highlights the fact that the majority of the negative subjects are truly negative, while positive obtained results should be confirmed by other methods such as culture or serology. The NPV is also clinically significant, because it can prevent unnecessary IAP and consequently will diminish the development of antibiotic resistance and anaphylaxis. The low sensitivity in our study (72.7%) mainly was resulted from six false negative results for real-time PCR (Table 2). The discrepancy between the results from culture and real-time PCR can be attributed to incorrect sampling, cross-contamination between specimens during assay, low sensitivity of the commercial kit, and light initial bacterial colonization in the rectum or vagina. Although such false-negative results have occurred in other previous studies, they have reported higher sensitivity than our findings (Bergseng et al., 2007; Gavino & Wang, 2007). In all cases, we evaluated the culture results after an initial enrichment of the samples in the selective broth. This process supports the growth of small numbers of bacteria in condition of light colonization. Despite culture, real-time PCR was conducted on DNA samples obtained from direct inoculation of swabs in PBS buffer. It can reasonably explain the reduced sensitivity of real-time PCR method in comparsion with pre-enriched culture. In support of this hypothesis, Goodrich et al., (Goodrich & Miller, 2007) compared the licensed real-time PCR tests with the standard culture method. Interestingly, they found that performance of real-time PCR on rectal/vaginal swabs enriched in selective broth would increase the sensitivity value 92.5% to 100% relative to culture. Although enrichment in broth medium before DNA extraction, prolongs the time of assay, this modification improves the sensitivity of real-time PCR and facilitates bacterial identification and antimicrobial susceptibility testing. Although various studies have not reported resistance to first-line beta-lactam antibiotics (e.g. penicillin and ampicillin) (Berkowitz, Regan, & Greenberg, 1990; Garland et al., 2011; Quiroga, Pegels, Oviedo, Pereyra & Vergara, 2008); however, others have found rising number of isolates with elevated minimum inhibitory concentration (MIC) to penicillin and ampicillin. For instance, 14 isolates in Japan and 0.2% of invasive isolates in the USA had raised MICs to the above-mentioned beta-lactams, through 1995-2005 and 1999-2005, respectively (Kimura et al., 2008). Moreover, modifications in penicillin-binding proteins have been demonstrated in all isolates from Japan and four isolates from the USA (Dahesh et al., 2008). In Ardabil (northwest of Iran), Jannati et al. (2012) examined 56 GBS isolates with the disk diffusion and E-test methods and found only one isolate (1.7%) with reduced susceptibility (MIC: 0.25 µg/ml) to penicillin. During 1999-2007, resistance to erythromycin and clindamycin in various geographic regions has been estimated 3%-54% and 1%-43%, respectively (Garland et al., 2011; Heelan et al., 2004; Janapatla, Ho, Yan, Wu & Wu, 2008; Matsubara et al., 2001; Simoes et al., 2004). In Italy, Lambias et al., (2012) assessed 879 GBS isolates and detected the rising trend of resistance to erythromycin and clindamycin (from 16.5% in 2005 to 69.9% in 2008). In spite of the reasonable frequency rate of penicillin resistance among our GBS isolates, the prevalence of resistance to erythromycin (an alternative antibiotic for women allergic to penicillin) was higher in the present research than other studies. We were the first to report the antimicrobial susceptibility pattern of the a few GBS population in Lorestan region using the disk diffusion method. However, MIC determination of isolates for some first and second-line antibiotics was virtually impossible, due to limited accessibility to reliable antibiotic powders or E-test strips. Generally, the results of the current and previous studies indicate the relatively high frequency of GBS carriers among Iranian pregnant women, as well as, Khorramabad. Unfortunately, despite the programs and policies of CDC, regarding routine screening of pregnant women for GBS, these strategies are commonly ignored in most areas of Iran, as well as Lorestan region.

## 5. Conclusion

Health authorities dealing with maternal and newborn health care are recommended to conform global or national policies for screening of pregnant women. In addition, all GBS strains isolated from various parts of the country should be regularly examined in terms of susceptibility, and uniform regional/national prevention and treatment guidelines be adopted and issued. Based on our findings, penicillin can be the antibiotic of choice for IAP at least in our region. In penicillin-allergic mothers, vancomycin can be as an alternative antibiotic.
